# *In Vitro* Studies on the Antioxidant Property and Inhibition of *α*-Amylase, *α*-Glucosidase, and Angiotensin I-Converting Enzyme by Polyphenol-Rich Extracts from Cocoa *(Theobroma cacao)* Bean

**DOI:** 10.1155/2014/549287

**Published:** 2014-09-08

**Authors:** Ganiyu Oboh, Ayokunle O. Ademosun, Adedayo O. Ademiluyi, Olasunkanmi S. Omojokun, Esther E. Nwanna, Kuburat O. Longe

**Affiliations:** Functional Foods and Nutraceuticals Unit, Biochemistry Department, Federal University of Technology, PMB 704, Akure 340252, Nigeria

## Abstract

*Background*. This study sought to investigate the antidiabetic and antihypertensive mechanisms of cocoa (*Theobroma cacao*) bean through inhibition of *α*-amylase, *α*-glucosidase, angiotensin-1 converting enzyme, and oxidative stress. *Methodology*. The total phenol and flavonoid contents of the water extractable phytochemicals from the powdered cocoa bean were determined and the effects of the extract on *α*-amylase, *α*-glucosidase, and angiotensin-1 converting enzyme activities were investigated *in vitro*. Furthermore, the radicals [1,1-diphenyl-2 picrylhydrazyl (DPPH), 2,2..-azino-bis(3-ethylbenzthiazoline-6-sulphonic acid) (ABTS), hydroxyl (OH), and nitric oxide (NO)] scavenging ability and ferric reducing antioxidant property of the extract were assessed. *Results*. The results revealed that the extract inhibited *α*-amylase (1.81 ± 0.22 mg/mL), *α*-glucosidase (1.84 ± 0.17 mg/mL), and angiotensin-1 converting enzyme (0.674 ± 0.06 mg/mL [lungs], 1.006 ± 0.08 mg/mL [heart]) activities in a dose-dependent manner and also showed dose-dependent radicals [DPPH (16.94 ± 1.34 mg/mL), NO (6.98 ± 0.886 mg/mL), OH (3.72 ± 0.26 mg/mL), and ABTS (15.7 ± 1.06 mmol/TEAC*·*g] scavenging ability. *Conclusion*. The inhibition of *α*-amylase, *α*-glucosidase, and angiotensin-1 converting enzyme activities by the cocoa bean extract could be part of the possible mechanism by which the extract could manage and/or prevent type-2 diabetes and hypertension.

## 1. Introduction

The studies of cocoa and their related products have become an area of interest owing to their health-promoting properties. In recent years, cocoa and cocoa products, namely, cocoa powder, dark chocolate, and cocoa liquor, have been shown to suppress atherosclerosis and reduce the risk of heart disease [1], increase dermal blood circulation, and decrease platelet activation, adhesion, and function, as well as function as cancer protective agent by inhibiting the proliferation of human cancer cells and also exerted hypoglycemic properties [2] owing to the presence of the phenolic compounds in it. Polyphenols have been researched for decades, mostly because of their antioxidant properties [3].

Dietary antioxidants sourced from the diet such as fruits and vegetables are capable of counteracting the damaging but normal effects of the physiological process of oxidation reactions that occur in animal tissue [4]. These antioxidants are considered beneficial because of their protective role against oxidative stress, which is involved in the pathogenesis of multiple diseases such as cardiovascular and cerebrovascular diseases [5]. Diabetes mellitus is a complex disease that is characterized by chronic hyperglycemia. The peculiar clinical features like excessive urination, thirst, weight loss. and secondary complications observed in diabetes mellitus are primarily an indication of the hyperglycemic state [6]. Type 1 diabetes results from inadequate synthesis of insulin by *β*-cells of the pancreas, while type II diabetes is characterized primarily by insulin resistance (a condition in which peripheral cells do not respond normally to insulin) or *β*-cell dysfunction [7].

Alpha-amylase is a prominent enzyme found in the pancreatic juice and saliva which breaks down large insoluble starch molecules into absorbable molecules [8]. On the other hand, mammalian *α*-glucosidase is an enzyme found in the mucosal brush border of the small intestine which catalyzes the end step of digestion of disaccharides that are abundant in human diet to its corresponding monosaccharide [9]. Effective means of lowering the levels of postprandial hyperglycemia have been offered by *α*-amylase and *α*-glucosidase inhibitors by delaying the breakdown of ingested carbohydrates in the small intestine thereby reducing the postprandial blood glucose excursion [10]. Several inhibitors of *α*-amylase and *α*-glucosidase have been isolated from medicinal plants to serve as an alternative drug with increased potency and less adverse effects than existing synthetic drugs [11].

Angiotensin-I converting enzyme (ACE) activity has been linked to hypertension. Inhibition of ACE is considered a useful therapeutic approach in the treatment of high blood pressure and dietary phenolic phytochemicals have shown promising potential while previous* in vitro* and* in vivo* animal and clinical studies have also indicated the potential of specific phenolic phytochemicals in hypertension management with absorption into the blood [12]. However, despite the documented antidiabetic [13] and antihypertensive potential of* Theobroma cacao *[14] no previous report has been given on the mechanism by which it exerts this effect.

## 2. Materials and Methods

### 2.1. Chemicals

All chemicals used were sourced from Sigma Corporation (St. Louis MO). Except stated otherwise, all the chemicals and reagents used are of analytical grade, while the water used was glass distilled.

### 2.2. Plant Material Collection and Preparation

Cocoa beans were bought at a local market in Ibule-soro (a suburb of Akure) near Federal University of Technology, Akure, Ondo State, Nigeria. The authentication of the bean was done at the Department of Crop, Soil and Pest Management, Federal University of Technology, Akure, Nigeria. Subsequently, the dried beans were then milled into fine powder. The aqueous extracts of the bean were prepared by soaking 5 g of the grinded samples in 100 mL of distilled water for 24 hrs at 37°C. The mixture was later filtered through Whatmann number 2 filter paper and centrifuged at 4000 rpm to obtain a clear supernatant which was then stored in the refrigerator for subsequent analysis [15].

### 2.3. Total Phenol Determination

The total phenol content was determined by mixing 0.2 mL of the sample extract with 2.5 mL 10% Folin-Cioalteau reagent (v/v) and 2.0 mL of 7.5% sodium carbonate was subsequently added. The reaction mixture was incubated at 45°C for 40 min, and the absorbance was measured at 765 nm using a spectrophotometer. Gallic acid was used as standard phenol; the total phenol content was subsequently calculated as gallic acid equivalent [16].

### 2.4. Total Flavonoid Determination

The total flavonoid content was determined by mixing 0.5 mL of appropriately diluted sample with 0.5 mL methanol, 50 *μ*L 10% AlC1_3_, 50 *μ*L 1 M Potassium acetate, and 1.4 mL distilled water and allowed to incubate at room temperature for 30 min. The absorbance of the reaction mixture was subsequently measured at 415 nm; quercetin is used as standard flavonoid. The total flavonoid content was subsequently calculated as quercetin equivalent. The nonflavonoid polyphenols were taken as the difference between the total phenol and total flavonoid content [17].

### 2.5. Enzyme Inhibition Assay

#### 2.5.1. Angiotensin I Converting Enzyme (ACE) Inhibition Assay

Appropriate dilution of the aqueous extract (0–500 *μ*L) and ACE solution (50 *μ*L, 4 mU) was incubated at 37°C for 15 min. The enzymatic reaction was initiated by adding 150 *μ*L of 8.33 mM of the substrate Bz - Gly - His - Leu in 125 mM Tris-HCl buffer (pH 8.3) to the mixture. After incubation for 30 min at 37°C, the reaction was arrested by adding 250 *μ*L of 1 M HCl. The Gly-His bond was then cleaved and the Bz-Gly produced by the reaction was extracted with 1.5 mL ethyl acetate. Thereafter the mixture was centrifuged to separate the ethyl acetate layer; then 1 mL of the ethyl acetate layer was transferred to a clean test tube and evaporated. The residue was redissolved in distilled water and its absorbance was measured at 228 nm [18]. The ACE inhibitory activity was expressed as percentage (%) inhibition.

#### 2.5.2. *α*-Amylase Inhibition Assay

This was measured using the dinitrosalicylic acid method as described by Worthington Biochemical Corporation 1978 [19]. Appropriate dilution of the pastes (500 *μ*L) and 500 *μ*L of 0.02 M sodium phosphate buffer (pH 6.9 with 0.006 M NaCl) containing pancreatic *α*-amylase (EC 3.2.1.1) (0.5 mg/mL) were incubated at 25°C for 10 min. Then, 500 *μ*L of 1% starch solution in 0.02 M sodium phosphate buffer (pH 6.9 with 0.006 M NaCl) was added to each tube. The reaction mixtures was incubated at 25°C for 10 min and stopped with 1.0 mL of dinitrosalicylic acid colour reagent. Thereafter, the mixture was incubated in a boiling water bath for 5 min and cooled to room temperature. The reaction mixture was then diluted by adding 10 mL of distilled water, and absorbance measured at 540 nm. The EC_50_ (the extract concentration inhibiting 50% of the *α*-amylase activity) of the pastes was calculated.

#### 2.5.3. *α*-Glucosidase Inhibition Assay

The extract (50 *μ*L) and 100 *μ*L of *α*-glucosidase solution (1.0 U/mL) in 0.1 M phosphate buffer (pH 6.9) was incubated at 25°C for 10 min. Then, 50 *μ*L of 5 mM p-nitrophenyl-*α*-D-glucopyranoside solution in 0.1 M phosphate buffer (pH 6.9) was added. The mixtures were incubated at 25°C for 5 min before reading the absorbance at 405 nm in the spectrophotometer. The *α*-glucosidase inhibitory activity was expressed as percentage inhibition. The EC_50_ of the pastes was calculated [20].

### 2.6. *In Vitro *Antioxidant Studies

#### 2.6.1. Determination of Reducing Property

The reducing property was determined by assessing the ability of the sample extract to reduce FeCl_3_ solution as described by Pulido et al., 2002 [21]. Briefly, appropriate dilutions (0–1.0 mL) were mixed with 2.5 mL 200 mm sodium phosphate buffer (ph 6.6) and 2.5 mL of 1% potassium ferricyanide. The mixtures were incubated at 50°C for 20 min. Thereafter, 2.5 mL 10% trichloroacetic acid was added and subsequently centrifuged at 650 rpm for 10 min. Then 5 mL of the resulting supernatant was mixed with equal volume of water and 1 mL of 0.1% ferric chloride. The absorbance was taken at 700 nm against a reagent blank.

#### 2.6.2. DPPH Free Radical Scavenging Assay

The free radical scavenging ability of the extract against DPPH (1,1-diphenyl-2picrylhdrazyl) free radical was evaluated as described by Gyamfi et al., 1999 [22]. Briefly, appropriate dilution of the extracts (0–500 *μ*L) was mixed with 1 mL, 0.4 mM methanolic solution containing DPPH radicals; the mixture was left in the dark for 30 min and the absorbance was taken at 516 nm. The DPPH free radical scavenging ability was subsequently calculated.

#### 2.6.3. ABTS^*·*+^ Radical Scavenging Ability Assay

The ABTS^*·*+^ (2,2′-azino-bis(3-ethylbenzthiazoline-6-sulphoic acid) scavenging ability of the extract was determined according to the method described by Re et al., 1999 [23]. The ABTS^*·*+^ was generated by reacting 7 mM ABTS aqueous solution with K_2_S_2_O_8_ (2.45 mmol/L, final concentration) in the dark for 16 hrs and adjusting the Abs 734 nm to 0.700 with ethanol. Thereafter, 200 *μ*L of appropriate dilution of the extract was added to 2.0 mL ABTS^*·*+^ solution and the absorbance was measured at 734 nm after 15 min. The trolox equivalent antioxidant capacity was subsequently calculated using trolox as the standard.

#### 2.6.4. Inhibition of the Fenton Reaction (Degradation of Deoxyribose)

The method of Halliwell and Gutteridge 1981 [24] was used to determine the ability of the extract to prevent Fe^2+^/H_2_O_2_ induced decomposition of deoxyribose. The extract (0–100 *μ*L) was added to a reaction mixture containing 120 *μ*L of 20 mM deoxyribose, 400 *μ*L of 0.1 M phosphate buffer, 40 *μ*L of 500 *μ*M FeSO_4_, and the volume were made up to 800 *μ*L with distilled water. The reaction mixture was incubated at 37°C for 30 min and the reaction was then stopped by the addition of 0.5 mL of 2.8% trichloroacetic acid (TCA). This was followed by addition of 0.4 mL of 0.6% thiobarbituric acid (TBA) solution. The tubes were subsequently incubated in boiling water for 20 min and the absorbance was measured at 532 nm in a spectrophotometer.

#### 2.6.5. Nitric Oxide Radical Scavenging Assay

The scavenging effect of the extract on nitric oxide (NO) radical was measured according to the method of Marcocci et al., 1994 [25]. Samples of 100–400 *μ*L of the oil extract were added in the test tubes to 1 mL of Sodium nitroprusside solution (25 mM) and tubes incubated at 37°C for 2 hours. An aliquot (0.5 mL) of the incubation was removed and diluted with 0.3 mL Griess reagent (1% sulphanilamide in 5% H_3_PO_4_ and 0.1% naphthlethylenediaminedihy drochloride). The absorbance of the chromophore formed was immediately read at 570 nm against distilled water as blank with catechin (50 *μ*g) used as standard. Results were expressed as percentage radical scavenging activity (RSA).

### 2.7. Lipid Peroxidation Assay

#### 2.7.1. Preparation of Tissue Homogenates

The rats were decapitated under mild diethyl ether anaesthesia; the pancreas was rapidly isolated, placed on ice, and weighed. This tissue was subsequently homogenized in cold saline (1/10, w/v) with about 10-up-and down strokes at approximately 1200 rev/min in a Teflon glass homogenizer. The homogenate was centrifuged for 10 min at 3000 ×g to yield a pellet that was discarded, and a low-speed supernatant (S1) that was kept for lipid peroxidation assay.

#### 2.7.2. Lipid Peroxidation and Thiobarbituric Acid Reactions

The lipid peroxidation assay was carried out using the modified method of Ohkawa et al., 1979 [26]. Briefly 100 *μ*L Sl fraction was mixed with a reaction mixture containing 30 *μ*L of 0.1 M pH 7.4 Tris-HCl buffer, extract (0–100 *μ*L) and 30 *μ*L of 250 *μ*M freshly prepared FeSO_4_ (the procedure was also carried out using 7 *μ*M sodium nitroprusside). The volume was made up to 300 *μ*L by water before incubation at 37°C for 1 hr. The reaction was developed by adding 300 *μ*L 8.1% Sodium doudecylsulphate (SDS) to the reaction mixture and this was subsequently followed by the addition of 600 *μ*L of acetic acid/HCl (pH 3.4) and 600 *μ*L 0.8% thiobarbituric acid (TBA). This mixture was incubated at 100°C for 1 hr and the thiobarbituric acid reactive species (TBARS) produced were measured at 532 nm. Subsequently, the lipid peroxidation was calculated as MDA produced (percentage of control).

### 2.8. Data Analysis

The results of three replicates were pooled and expressed as mean ± standard deviation (SD). One-way analysis of variance (ANOVA) and least significance difference (LSD) were carried out [27]. Significance was accepted at *P* ≤ 0.05. EC_50_ was determined using linear regression analysis

## 3. Results

The total phenolic content reported as gallic acid equivalent, total flavonoid content reported as quercetin equivalent, ABTS^∗^ scavenging ability reported as trolox equivalent, and ferric reducing antioxidant property reported as ascorbic acid equivalent as presented in Table 1 were 17.9 mg*·*GAE/100 g, 6.84 mg*·*QUE/100 g, 15.7 mmol/TEAC*·*g, and 14.92 mg/AAE*·*g, respectively.

As shown in Table 2, the cocoa bean aqueous extract scavenged DPPH, OH, and NO radicals in concentration-dependent manners, with EC_50_ values of 16.94 mg/mL, 3.72 mg/mL, and 6.98 mg/mL, respectively.

The antioxidant effect of the cocoa powder by interacting the aqueous extract with isolated rat pancreas in the presence of sodium nitroprusside (SNP) and Fe^2+^ as prooxidants was investigated. As shown in Table 3, the EC_50_ results show that the extract inhibits SNP (11.47 mg/mL) and Fe^2+^ (2.12 mg/mL) induced lipid peroxidation in rat's pancreas. In assessing the antihypertensive and antidiabetic potentials of the extract, the results revealed that the bean extracts inhibited angiotensin-I converting enzyme, *α*-amylase, and *α*-glucosidase activities. The EC_50_ values as presented in Table 3 were found to be 1.81 mg/mL (*α*-amylase), 1.84 mg/mL (*α*-glucosidase), 0.674 mg/mL (ACE in the lungs), and 1.01 mg/mL (ACE in the heart).

## 4. Discussion

Previous studies on the health benefits of cocoa have primarily focused on its effects on the risk of cardiovascular diseases and reduction of blood glucose levels [28], with a dearth of information on the possible mechanisms of action. The inhibition of *α*-amylase slows down the breakdown of starch to disaccharide, while the inhibition of *α*-glucosidase slows down the breakdown of the disaccharides to the simple monosaccharide, glucose, thereby reducing the amount of glucose absorbed into the blood stream [10]. Angiotensin-1 converting enzyme (ACE) cleaves angiotensin I which is a decapeptide to produce angiotensin II, an octapeptide and potent vasoconstrictor that has been identified as a major factor in hypertensive conditions [29]. Therefore, ACE inhibitors have been widely considered to prevent angiotensin II production in cardiovascular diseases, and utilized in clinical applications since the discovery of ACE inhibitors in snake venom [29]. The inhibition of *α*-amylase and *α*-glucosidase activities by the cocoa powder aqueous extracts could have contributed to its use in the management of diabetes, thereby slowing down the breakdown of starch to disaccharide. The extract inhibited both *α*-glucosidase and *α*-amylase in a dose-dependent manner; pointing to its potential of being of great pharmaceutical importance, in addressing some of the side effects (like flatulence and abdominal distention) associated with the drugs (Acarbose, Miglitol, and Voglibose) presently used for the management of diabetes. Furthermore, the dose-dependent ACE inhibitory activity of the cocoa powder aqueous extract reveals that this could explain the likely mechanism for its use in the treatment of hypertension and possibly address the side effects of some class of antihypertensive drugs (diuretics, beta blockers, and synthetic ACE inhibitors) used in managing hypertension.

Polyphenols have received wide attention because of their antioxidant properties which refers to their ability to prevent damage from reactive oxygen species through free radical scavenging or prevent the generation of these species by iron chelation as well as bind and inhibit the enzymes *α*-amylase and *α*-glucosidase [30]. The antioxidant activity of fruits and vegetables significantly increases with the increase in polyphenol content in it [31]. The total phenol and flavonoid content of this cocoa bean extract was found to be 17.9 mg*·*GAE/100 g and 6.84 mg*·*QUE/100 g, respectively. This finding was in agreement with previous study which demonstrated that high flavonoid (a class of polyphenol) content of cocoa extract correlated with its strong antioxidant capacity [28].

The free radical scavenging ability of this extract was also studied making use of moderately stable nitrogen-centred radical species-ABTS radical [32]. The result obtained here agrees with the phenolic content in earlier research articles, where correlations were reported between phenolic content and antioxidant capacity of some plant foods [33]. The extract was found to also demonstrate strong free radical scavenging abilities as exemplified by their scavenging activity of moderately stable ABTS^*·*+^, NO, OH^−^, and DPPH radicals* in vitro*. However, there is an agreement between the ABTS^*·*+^, NO, OH^−^, and DPPH free radical scavenging ability, which agrees with earlier findings where plant antioxidant properties (free radical scavenging ability) correlates with their phenolic content [34]. A study showed that decrease in antioxidant capacity in cocoa beverages stored at different temperature and time was due to loss of its phenolic content [35].

Antioxidants neutralize the electrical charges on free radicals and prevent them from taking electrons from other molecules [36]. They are needed to prevent the formation and oppose the actions of reactive oxygen and nitrogen species, which are generated* in vivo* and cause damage to DNA, lipids, proteins, and other biomolecules. Antioxidants are naturally powerful substances that help slow the aging process, fight diseases such as diabetes mellitus and hypertension, and prevent some deadly and incurable diseases like cancer [37]. The protective ability of the extract against Sodium nitroprusside and Fe^2+^ induced lipid peroxidation in cultured rats' pancreas assessed revealed that incubation of the pancreas tissues in the presence of Fe^2+^ caused a significant (*P* < 0.05) increase in the MDA content (181%). Likewise, incubation of the pancreas tissue in the presence of sodium nitroprusside caused a significant (*P* < 0.05) increase in the MDA content (249%). These findings agree with earlier reports, where phenolics had been reported to be potent inhibitors of lipid peroxidation in several animal tissues [38]. Sodium nitroprusside; a component of antihypertensive drugs is known to cause cytotoxicity through the release of cyanide and/or nitric oxide (NO). NO is a universal neuronal messenger in the central nervous system and acts independently, it may also cause neuronal damage in cooperation with other reactive oxygen species (ROS) [39]. It could be suggested that the higher ACE inhibitory ability of the aqueous extracts of the cocoa powder aqueous extract is due to its higher NO∗ scavenging ability. However, we advise that further work should be done to isolate the phytoconstituents in the cocoa bean which may be responsible for eliciting the biological activities. Also,* in vitro* works do not automatically translate to human body response; hence, we suggest that further* in vivo* work should be carried out.

## 5. Conclusion

Conclusively, cocoa bean is rich in phenolic compounds and exhibits a high antioxidant activity. Its ingestion as a functional food lowers plasma glucose level by inhibiting *α*-amylase and *α*-glucosidase and could be of importance in the management of diabetes mellitus as it also addresses some side effects associated with synthetic antidiabetic drugs. In addition, cocoa seed extract inhibition of ACE also explains the likely mechanism for its use in the treatment of hypertension. Hence, cocoa bean ingestion will improve the quality of life of hypertensive patients and the diabetics.

## Figures and Tables

**Table 1:** The total phenol content reported as gallic acid equivalent, total flavonoid content reported as quercetin equivalent, ABTS∗ scavenging ability reported as trolox equivalent antioxidant capacity, and ferric reducing antioxidant property reported as ascorbic acid equivalent of aqueous extract of cocoa bean.

Parameter	Value (unit)
Total phenol	17.9 ± 0.96 (mg*·*GAE/100 g)
Total flavonoid	6.84 ± 0.10 (mg*·*QUE/100 g)
ABTS∗ scavenging ability	15.7 ± 1.06 (mmol/TEAC*·*g)
Ferric reducing antioxidant property	14.92 ± 0.82 (mg/AAE*·*g)

Values represent means ± standard deviation of triplicate readings.

**Table 2:** EC_50_ values of DPPH, OH, and NO radical scavenging ability of aqueous extract of cocoa bean.

Parameter	EC_50_ value (mg/mL)
DPPH radical scavenging ability	16.94 ± 1.34
OH radical scavenging ability	3.72 ± 0.26
NO radical scavenging ability	6.98 ± 0.88

Values represent means ± standard deviation of triplicate readings.

**Table 3:** EC_50_ values of SNP and Fe^2+^ induced lipid peroxidation in rat's pancreas, angiotensin-I converting enzyme inhibitory activity in the lungs and heart, and *α*-amylase and *α*-glucosidase inhibitory activity of aqueous extract of cocoa bean.

Parameter	EC_50_ value (mg/mL)
SNP induced lipid peroxidation	11.47 ± 1.07
Fe^2+^ induced lipid peroxidation	2.12 ± 0.10
ACE (lungs)	0.674 ± 0.06
ACE (heart)	1.006 ± 0.08
*α*-amylase	1.81 ± 0.22
*α*-glucosidase	1.84 ± 0.17

Values represent means ± standard deviation of triplicate readings.
